# Weightbearing after combined medial and lateral plate fixation of AO/OTA 41-C2 bicondylar tibial plateau fractures: a biomechanical study

**DOI:** 10.1186/s12891-022-05024-2

**Published:** 2022-01-25

**Authors:** Sorawut Thamyongkit, Pooyan Abbasi, Brent G. Parks, Babar Shafiq, Erik A. Hasenboehler

**Affiliations:** 1 Department of Orthopaedic Surgery, The Johns Hopkins University/Johns Hopkins Bayview Medical Center, 4940 Eastern Ave., #A667, Baltimore, MD 21224-2780 USA; 2 Chakri Naruebodindra Medical Institute, Faculty of Medicine Ramathibodi Hospital, Mahidol University, Samut Prakan, Thailand; 3 Department of Orthopaedic Surgery, MedStar Union Memorial Hospital, Baltimore, MD USA

**Keywords:** Biomechanics, Bone plates, Fracture fixation, Tibial fractures, Tibial plateau, Weightbearing

## Abstract

**Background:**

Combined medial and lateral plate fixation is recommended for complex tibial plateau fractures with medial fragments or no cortical bone contact. Although such fixation is adequate to resist forces during range of motion, it may be insufficient to support immediate postoperative weightbearing. Here, we analyzed displacement, stiffness, and fixation failure during simulated full weightbearing of bicondylar tibial plateau fractures treated with combined medial and lateral locking plate fixation.

**Methods:**

We used 10 fresh-frozen adult human cadaveric tibias and mated femurs. Osteotomies were performed with an oscillating saw and cutting template to simulate an AO Foundation and Orthopaedic Trauma Association (AO/OTA) 41-C2 fracture (simple articular, multifragmentary metaphyseal fracture). Specimens were anatomically reduced and stabilized with combined medial and lateral locking plates (AxSOS, Stryker, Mahwah, NJ). Specimens were loaded axially to simulate 4 weeks of walking in a person weighing 70 kg. The specimens were cyclically loaded from 200 N to a maximum of 2800 N. Then, if no failure, loading continued for 200,000 cycles. We measured displacement of each bone fragment and defined fixation failure as ≥5 mm of displacement. Construct stiffness and load at failure were calculated. Categorical and continuous data were analyzed using Chi-squared and unpaired t-tests, respectively.

**Results:**

Mean total displacement values after 10,000 loading cycles were as follows: lateral, 0.4 ± 0.8 mm; proximal medial, 0.3 ± 0.7 mm; distal medial, 0.3 ± 0.6 mm; and central 0.4 ± 0.5 mm. Mean stiffness of the construct was 562 ± 164 N/mm. Fixation failure occurred in 6 of 10 specimens that reached 5 mm of plastic deformation before test completion. In the failure group, the mean load at failure was 2467 ± 532 N, and the mean number of cycles before failure was 53,155. After test completion, the greatest displacement was found at the distal medial fracture site (2.3 ± 1.4 mm) and lateral fracture site (2.2 ± 1.7 mm).

**Conclusions:**

Although combined medial and lateral plate fixation of complex tibial plateau fractures provides adequate stability to allow early range of motion, immediate full weightbearing is not recommended.

## Introduction

Fractures of the tibial plateau are often caused by high-energy mechanisms and involve major injury to bone and soft tissue. Complication rates are high because of the diversity and complexity of fracture patterns and the thin soft-tissue envelope around the knee [1–3]. Therefore, treatment and treatment planning are extraordinarily challenging for these fractures [4]. Goals of surgical treatment of displaced bicondylar tibial plateau fractures include anatomic reduction of the articular surface, restoration of alignment in the coronal and sagittal planes, and stable fixation to enable early knee motion. To provide the stability needed to safely permit early range of motion (ROM) and basic strengthening exercises, many surgeons use combined medial and lateral plating. Many surgeons also use delayed-weightbearing protocols after fixation of bicondylar tibial plateau fractures with metaphyseal comminution because of concern about fixation failure or articular displacement. In 2007, Higgins et al. [5] performed a biomechanical study comparing dual plate fixation (medial and lateral) with lateral locked plate fixation alone in bicondylar fractures. Specimens were subjected to cyclic loading, simulating forces encountered during early postoperative range of motion (ROM) exercises or activities of daily living [5]. Dual plate fixation produced significantly less subsidence compared with locked lateral plate fixation alone. However, lateral locking plate fixation alone was deemed sufficient in cases with a large medial fragment with good cortical contact and no coronal fracture line. Otherwise, dual plating was recommended. In biomechanical studies [5–7], combined medial and lateral plate fixation effectively resisted forces encountered during ROM. However, none of these studies reported on fracture displacement or loss of fixation when implementing an early/immediate (at 2 weeks) full weightbearing protocol.

To our knowledge, no biomechanical study has simulated full weightbearing in bicondylar tibial plateau fractures treated with combined medial and lateral plate fixation. Our purpose was to analyze displacement, stiffness, and fixation failure during simulated full weightbearing of bicondylar tibial plateau fractures treated with combined medial and lateral locking plate fixation. We simulated full weightbearing in AO Foundation and Orthopaedic Trauma Association (AO/OTA) 41-C2 fractures (simple articular, multifragmentary metaphyseal fracture). Our model represents a simple articular pattern without articular depression but with metaphyseal comminution necessitating dual plating. We hypothesized that combined medial and lateral plate fixation, although adequate to resist forces during ROM, would be insufficient for immediate full weightbearing.

## Materials and methods

We conducted a biomechanical study using 10 fresh-frozen adult human cadaveric tibias and mated femurs. All specimens were screened via radiography for varus/valgus deformities, previous fractures, bone defects, and osteolysis. Bone mineral density (BMD) measurements were obtained from the proximal femur using a dual-energy x-ray absorptiometry scanner (Lunar iDXA, GE Healthcare, Madison, WI, USA). The mean (± standard deviation) BMD of all specimens was 1.1 ± 0.1 g/cm^3^. There was no significant difference in mean BMD of specimens in the failure group versus the non-failure group (Table 1).

**Table 1:** Comparison of bone mineral density and stiffness between the human cadaveric tibia specimens that withstood loading up to 200,000 cycles and those that failed

Parameter	Mean ± Standard Deviation		*P*
	Failure group^a^ (*N* = 6)	Non-failure group (*N* = 4)	
Bone mineral density, g/cm^3^	1.1 ± 0.1	1.1 ± 0.1	0.90
Stiffness, N/mm	616 ± 81	482 ± 236	0.23

^a^Six specimens reached 5 mm of plastic deformation before test completion: 4 specimens failed at 2800 N, 1 failed at 2000 N, and 1 failed at 1600 N

### Specimen preparation

**Fig. 1:**
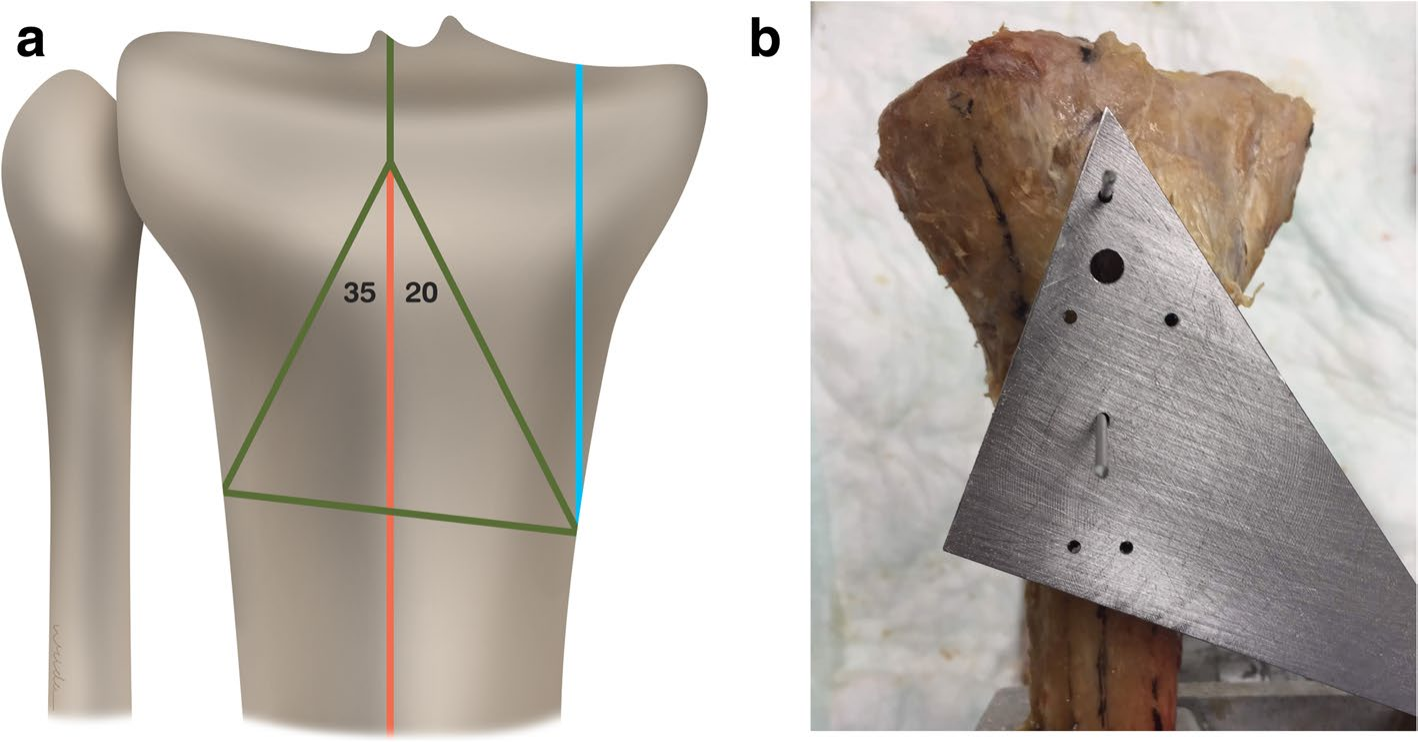
**a**: Illustration showing the osteotomy positions and angles used to recreate an AO Foundation and Orthopaedic Trauma Association 41-C2 fracture (simple articular, multifragmentary metaphyseal fracture) in a human cadaveric specimen. (Green line, cutting line as in the study by Gösling et al. [8] Blue line, additional medial cutting line. Red line, tibial axis.) **b** Template used to perform the osteotomies. The template was fitted to the tibia, and K-wires were fitted along the tibial axis and used to secure the template and assure standardized osteotomies

Specimens were prepared according to the methods of Gösling et al. [8], modified by the addition of a vertical fracture line in the medial fragment (Fig. 1a). A cutting template was designed to recreate standardized osteotomies (Fig. 1b), which were performed with an oscillating saw. To achieve exact positioning, we fitted the template to a perpendicular orientation wire. The first cut was directed from a point 2 cm distal to the intercondylar eminence to the medial cortex with a 20° angle to the tibial axis. A second cut was directed toward the lateral cortex with a 35° angle to the tibial axis. A third cut was made along the connecting line between the most distal medial and lateral cortical intersection. The final cut was a sagittal cut of the medial fragment parallel to the tibial axis to simulate a small medial fragment. We modified the fracture pattern to simulate an injury that lacks articular depression (AO/OTA 41C2) but has metaphyseal instability necessitating dual fixation. All fracture models were created by 1 fellowship-trained orthopaedic trauma surgeon.

### Reduction and fixation

**Fig. 2:**
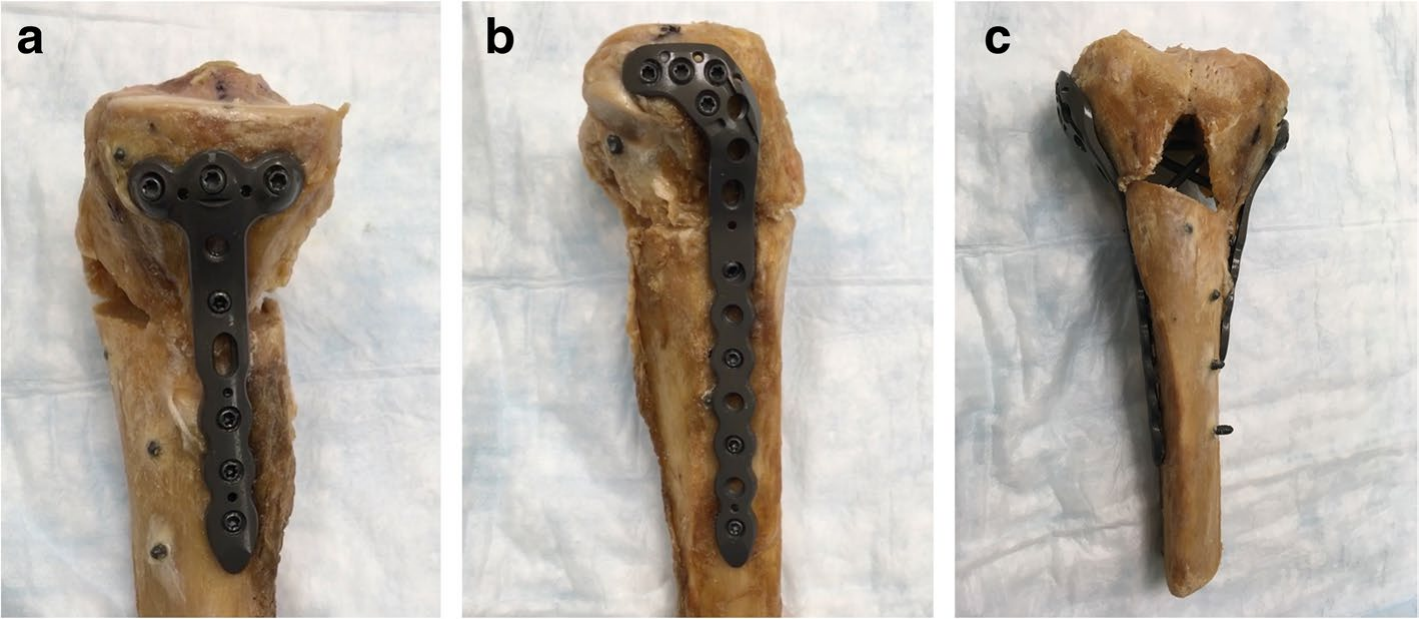
**a–c**. **a** Medial, **b** lateral, and **c** anterior view of a fresh-frozen human cadaveric tibia specimen after anatomical reduction and combined medial and lateral plate fixation with locking screws. The tibial fracture was reduced and fixed with a 6-hole, 4.0-mm proximal medial tibial plate (AxSOS, Stryker, NJ) for the medial side and a 6-hole, 4.0-mm proximal lateral tibial plate (AxSOS, Stryker)

All specimens were anatomically reduced under direct visualization. A K-wire was placed for temporary fixation. Subsequently, combined medial and lateral fixation was applied using 6-hole, 4.0-mm proximal medial and lateral tibial plates (AxSOS, Stryker, NJ, USA). On the medial side, the plate was secured with the proximal 3 subchondral screws, 3 screws in the shaft, and the angled midportion screw. On the lateral side, the plate was secured with 4 proximal screws, 3 screws in the shaft, and the 2 angled screws in the middle. All screws were locking (Fig. 2).

### Biomechanical testing

**Fig. 3:**
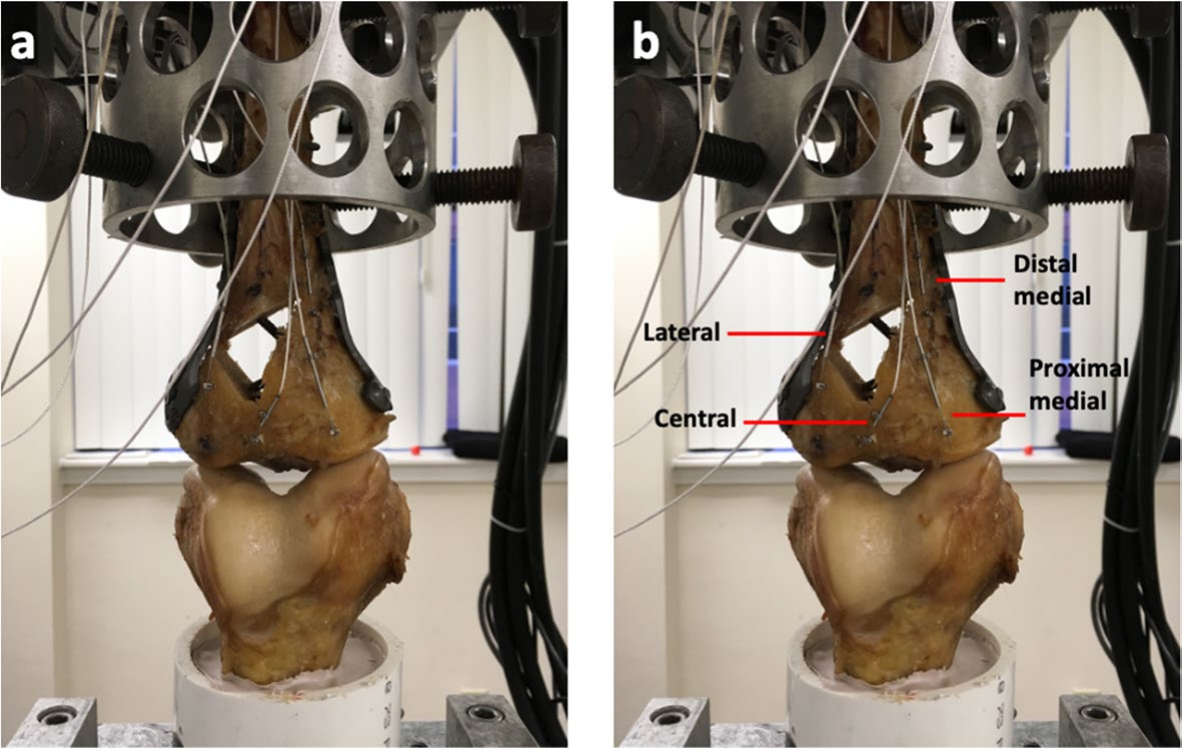
**a** Tibia and femur human cadaveric specimens mounted on the material testing frame (MTS 858 Mini-Bionix; MTS Systems, Eden Prairie, MN, USA). The femur was mounted in an inverted position in a 7.6-cm-diameter polyvinyl chloride pipe and stabilized with polyester resin. Femoral condyles were leveled horizontally for proper axial loading. **b** Four differential variable reluctance transducers sensors were mounted across the fracture sites (proximal medial, distal medial, center, and lateral fragments)

The femur was mounted in an inverted position in a 7.6-cm-diameter polyvinyl chloride pipe and stabilized with polyester resin. Femoral condyles were leveled horizontally for proper axial loading. Subsequently, the tibia was mounted in a cylindrical metal clamp, which was later attached to the loading piston of the material testing frame (MTS 858 Mini-Bionix; MTS Systems, Eden Prairie, MN, USA). This loading model simulated the normal load distribution of each pair of specimens with the joint line parallel to the floor [9, 10]. Four MDVRT-9 differential variable reluctance transducer sensors (Lord MicroStrain, Williston, VT) were mounted across the fracture sites (Fig. 3). Displacement between fragments was measured in millimeters at 4 fracture sites: lateral, central, proximal medial, and distal medial.

Specimens were then placed on the MTS loading frame and cyclically loaded between 20 N (minimum) and 200 N (maximum) for 2000 cycles at a frequency of 3 Hz. Load was then incrementally increased by 200 N to a maximum of 2800 N, each load increment being run for 2000 cycles. Fixation failure was defined as ≥5 mm of displacement of the fracture fragments at any fracture site. If no failure was observed, the specimen was incrementally loaded to 2800 N for a total of 200,000 cycles to simulate 4 weeks of walking [11]. Studies have established that healthy adults take approximately 5000 to 7000 steps per day [11–13], and the forces transmitted across the knee joint during normal walking are 2 to 3 times body weight [12, 14]. Thus, we used this testing protocol to simulate approximately 4 weeks of walking in a healthy person weighing 70 kg. The parameter of interest was displacement of bone fragments at the proximal medial, distal medial, center, and lateral fracture sites in response to the applied load. The initial stiffness of the entire construct was calculated at the 100th cycle for all specimens.

### Statistical analysis

According to Horwitz et al. [15], the sample size needed to detect a 2-mm difference with a power of 80% and a significance level of 5% was 6 specimens. Categorical and continuous data were analyzed using Chi-squared and unpaired t-tests, respectively. *P* < 0.05 was considered to be significant.

## Results

The mean initial stiffness of the construct was 562 ± 164 N/mm. All combined medial and lateral locking plate constructs maintained the integrity of the fixation in cyclic loading to 1000 N after 10,000 cycles. Mean total displacement values after 10,000 cycles were as follows: lateral, 0.4 ± 0.8 mm; proximal medial, 0.3 ± 0.7 mm; distal medial, 0.3 ± 0.6 mm; and central 0.4 ± 0.3 mm (Table 2).

**Table 2:** Displacement under cyclic loading at 1000 N after 10,000 cycles and after test completion in 10 fresh-frozen adult human cadaveric tibias with simulated AO/OTA 41-C2 bicondylar tibial plateau fractures (simple articular, multifragmentary metaphyseal fractures) treated with combined medial and lateral plate fixation

Specimen No.	Displacement after 10,000 cycles (mm)				Displacement at the end of testing (mm)^a^			
	Lateral	Proximal Medial	Distal Medial	Central	Lateral	Proximal Medial	Distal Medial	Central
1	0.1	0	0	0	1.9	5.0^b^	1.5	1.5
2	0	0	0	0.1	4.3	0.8	5.0^b^	0.2
3	0.1	0	0.2	0.4	4.8	1.6	1.7	0.5
4	0	0	0.1	0.1	2.8	0.2	5.0^b^	0.1
5	0.2	0.1	0.1	0.5	1.1	2.9	4.4	0.9
6	0	0	0	0	5.1^b^	0.2	1.9	0
7	2.7	2.3	2	1.6	5.3^b^	4.2	4.3	1.3
8	0.4	0.2	0.1	0.8	5.0^b^	1.7	1.8	0.5
9	0.1	0	0	0.2	0.3	0.8	2.9	0.9
10	0.1	0	0	0.3	0.4	0.1	0.1	0.4
Mean (SD)	0.4 (0.8)	0.3 (0.7)	0.3 (0.6)	0.4 (0.5)	2.2 (1.7)	1.4 (1.3)	2.3 (1.4)	0.6 (0.5)

*AO/OTA* AO Foundation and Orthopaedic Trauma Association; *SD* Standard deviation

^a^Displacement either after 200,000 cycles or at failure

^b^Displacement at failure

Four specimens withstood loading up to 200,000 cycles. The other 6 specimens reached 5 mm of plastic deformation before completing the test: 4 specimens failed at 2800 N, 1 failed at 2000 N, and 1 failed at 1600 N. The mean cyclic load at failure was 2467 ± 532 N, and the mean number of cycles to failure was 53,155 (Table 3). At the end of testing (either failure or after 200,000 cycles), mean displacement was greatest at the distal medial fracture site (2.3 ± 1.4 mm) and lateral fracture site (2.2 ± 1.7 mm). No significant difference was found in stiffness between the failure and non-failure groups (Table 1).

**Table 3:** BMD, stiffness, cycles to failure, and load at failure in10 fresh-frozen adult human cadaveric tibias with simulated AO/OTA 41-C2 bicondylar tibial plateau fractures (simple articular, multifragmentary metaphyseal fractures) treated with combined medial and lateral plate fixation

Specimen No.	BMD (g/cm3)	Stiffness (N/mm)	Cycles to failure	Load at failure (N)
1	0.9	551	54,411	2800
2	1.1	697	75,819	2800
3^a^	1	514	NA	NA
4	1	576	43,575	2800
5^a^	1.2	152	NA	NA
6	1.1	712	111,884	2800
7	1.2	646	14,851	1600
8	1.1	514	18,387	2000
9^a^	1	556	NA	NA
10^a^	1.1	708	NA	NA
Mean (SD)	1.1 (0.1)	562 (156)	53,155 (3349)	2467 (485)

*BMD* Bone mineral density, *NA* Not applicable, *SD* Standard deviation

^*^Specimens withstood loading up to 200,000 cycles

## Discussion

The purpose of our biomechanical study was to assess the ability of combined medial and lateral locked plate fixation of bicondylar tibial plateau fractures to resist fracture displacement during simulated full weightbearing. We subjected 10 specimens with simulated bicondylar tibial plateau fractures (AO/OTA 41C2) to repetitive loading using a previously described method. None of the 10 specimens experienced fixation failure after increasing the load to 1000 N at 10,000 cycles. Only small displacements (0–2.7 mm) were observed at this point. These results are consistent with those of a study on dual plate fixation of tibial plateau fractures [5]. However, only 4 of our specimens withstood maximum load testing (2800 N and 200,000 cycles). Six specimens failed (> 5 mm of displacement) before test completion. These findings support postoperative protocols delaying full weightbearing during the early postoperative period until some bony healing has occurred. Immediate, full weightbearing of bicondylar tibial plateau fractures, even in those with simple articular splits, may be too aggressive despite combined medial and lateral plate fixation [16]. Malunion, nonunion, and even fixation failure remain concerns with immediate weightbearing. In a web-based survey of 111 Dutch orthopaedic trauma surgeons, only 12% of surgeons recommended immediate weightbearing, with most (56%) recommending initiating weightbearing 6 weeks postoperatively [17]. Common protocols recommend nonweightbearing for 6 weeks or more, followed by progressive partial weightbearing for 4–6 weeks after visible callus is evident on follow-up radiographs [18]. The AO recommendations also support toe-touch weightbearing for 6–12 weeks to avoid loss of reduction [19].

However, restricting weightbearing requires patient compliance and greater energy expenditure compared with full-weightbearing walking [20, 21]. This need for greater effort may reduce patients’ compliance with rehabilitation protocols. Immediate, full weightbearing after locking plate osteosynthesis for partial articular proximal tibial fractures has been studied and recommended [22–24]. Thewlis et al. [16] reported on the functional outcomes after surgery of 17 patients with either complete or incomplete articular fractures (AO types 41-B and C). Their results showed no association between weightbearing and patient functional outcomes at 1 year. Williamson et al. [24] showed that patients who progressed to immediate, full weightbearing after open reduction internal fixation neither lost reduction nor reported worse functional outcomes at 1 year compared with patients who maintained partial weightbearing for 6 weeks. Because patients self-regulate their weightbearing when allowed early, full, earlier weightbearing may be safe [16, 24]. Although these results may appear to support an accelerated weightbearing protocol after open reduction and internal fixation of complex tibial plateau fractures, until now no biomechanical study has been published to support these results.

To our knowledge, ours is the first study to simulate full weightbearing after bicondylar tibial plateau fracture (AO/OTA 41-C2) fixation with dual plates. Our specimen testing protocol differed from previous studies in small but important ways. Because tibiofemoral contact forces range from 2 to 3 times body weight [14], we decided to use greater forces of cyclic loading than were used in previous studies [5, 8, 15]. When simulating 4 weeks of early full weightbearing, we used a 2800-N load to represent force transmitted across the knee during walking in a person weighing 70 kg. In this testing protocol, we assumed that no sufficient bony callus formed before 4 weeks and that an active adult loads each limb approximately 50,000 times per week [11]. results provide evidence that may help improve postoperative rehabilitation instructions and better define early weightbearing protocols. The fracture models developed by Horwitz et al. [15] and Gösling et al. [8] have been used in several biomechanical studies of bicondylar tibial plateau fractures. However, these models do not address the small size of the medial fragment, which requires thorough evaluation and appropriate fixation. The fracture line of the medial fragment can also vary in the coronal plane and commonly results in a posteromedial fragment [25]. This topic is important for future study of biomechanical stability of the posteromedial fragment in bicondylar tibial plateau fracture fixation.

Our study has several limitations. First, the force applied to the tibial plateau during weightbearing depends on an individual’s body weight and degree of knee flexion. Therefore, some patients may be able to tolerate early, full weightbearing and others may not. Second, a cadaveric study can mimic in vivo bone behavior under loading, but the absence of soft tissue and healing affects fragment stability. Intact surrounding soft tissue or degree of soft tissue injury might increase the stability of fracture in vivo. Third, we used titanium locking compression plates. Construct strength and stiffness might be increased with the use of stainless-steel implants. A larger biomechanical study assessing the effect of stainless-steel versus titanium, as well as variations in BMD and simulated patient weight, may be helpful to determine in which patients early weightbearing may be indicated.

## Conclusion

Combined medial and lateral locking compression plate fixation of bicondylar tibial plateau fractures (AO/OTA 41-C2) does not offer adequate stability to allow immediate/early full weightbearing. Combined medial and lateral plate fixation offers sufficient stability to enable limited or partial weightbearing. Hence, full weightbearing should be postponed until sufficient bony healing has occurred to support bone fragments and prevent fracture displacement and failure.

## Data Availability

The datasets used and/or analyzed during the current study are available from the corresponding author on reasonable request.

## Abbreviations

AO/OTA: The AO Foundation and Orthopaedic Trauma Association
BMD: Bone mineral density
ROM: Range of motion

## References

[1] Andrews JR, Tedder JL, Godbout BP (1992). Bicondylar tibial plateau fracture complicated by compartment syndrome. Orthop Rev.

[2] Sharma N, Singh V, Agrawal A, Bhargava R (2015). Proximal tibial fractures with impending compartment syndrome managed by fasciotomy and internal fixation: a retrospective analysis of 15 cases. Indian J Orthop.

[3] Watson JT (1994). High-energy fractures of the tibial plateau. Orthop Clin N Am.

[4] Papagelopoulos PJ, Partsinevelos AA, Themistocleous GS, Mavrogenis AF, Korres DS, Soucacos PN (2006). Complications after tibia plateau fracture surgery. Injury.

[5] Higgins TF, Klatt J, Bachus KN (2007). Biomechanical analysis of bicondylar tibial plateau fixation: how does lateral locking plate fixation compare to dual plate fixation?. J Orthop Trauma.

[6] Bonyun M, Nauth A, Egol KA, Gardner MJ, Kregor PJ, McKee MD, Wolinsky PR, Schemitsch EH (2014). Hot topics in biomechanically directed fracture fixation. J Orthop Trauma.

[7] Jiang R, Luo CF, Wang MC, Yang TY, Zeng BF (2008). A comparative study of less invasive stabilization system (LISS) fixation and two-incision double plating for the treatment of bicondylar tibial plateau fractures. Knee.

[8] Gösling T, Schandelmaier P, Marti A, Hufner T, Partenheimer A, Krettek C (2004). Less invasive stabilization of complex tibial plateau fractures: a biomechanical evaluation of a unilateral locked screw plate and double plating. J Orthop Trauma.

[9] Hsu RW, Himeno S, Coventry MB, Chao EY (1990). Normal axial alignment of the lower extremity and load-bearing distribution at the knee. Clin Orthop.

[10] Bini SA, Chung CC, Wu SA, Hansen EN (2021). Tibial mechanical Axis is nonorthogonal to the floor in Varus knee alignment. Arthroplasty Today.

[11] Brumback RJ, Toal TR, Murphy-Zane MS, Novak VP, Belkoff SM (1999). Immediate weight-bearing after treatment of a comminuted fracture of the femoral shaft with a statically locked intramedullary nail. J Bone Joint Surg Am.

[12] Bassett DR, Wyatt HR, Thompson H, Peters JC, Hill JO (2010). Pedometer-measured physical activity and health behaviors in U.S. adults. Med Sci Sports Exerc.

[13] Tudor-Locke C, Johnson WD, Katzmarzyk PT (2009). Accelerometer-determined steps per day in US adults. Med Sci Sports Exerc.

[14] D'Lima DD, Fregly BJ, Patil S, Steklov N, Colwell CW (2012). Knee joint forces: prediction, measurement, and significance. Proc Inst Mech Eng [H].

[15] Horwitz DS, Bachus KN, Craig MA, Peters CL (1999). A biomechanical analysis of internal fixation of complex tibial plateau fractures. J Orthop Trauma.

[16] Thewlis D, Fraysse F, Callary SA, Verghese VD, Jones CF, Findlay DM, Atkins GJ, Rickman M, Solomon LB (2017). Postoperative weight bearing and patient reported outcomes at one year following tibial plateau fractures. Injury.

[17] van der Vusse M, Kalmet PHS, Bastiaenen CHG, van Horn YY, Brink PRG, Seelen HAM (2017). Is the AO guideline for postoperative treatment of tibial plateau fractures still decisive? A survey among orthopaedic surgeons and trauma surgeons in the Netherlands. Arch Orthop Trauma Surg.

[18] Arnold JB, Tu CG, Phan TM, Rickman M, Varghese VD, Thewlis D, Solomon LB (2017). Characteristics of postoperative weight bearing and management protocols for tibial plateau fractures: findings from a scoping review. Injury.

[19] Ruedi TP, Buickley RE, Moran CG (2000). AO principles of fracture management.

[20] Westerman RW, Hull P, Hendry RG, Cooper J (2008). The physiological cost of restricted weight bearing. Injury.

[21] Warren CG, Lehmann JF (1975). Training procedures and biofeedback methods to achieve controlled partial weight bearing: an assessment. Arch Phys Med Rehabil.

[22] Haak KT, Palm H, Holck K, Krasheninnikoff M, Gebuhr P, Troelsen A (2012). Immediate weight-bearing after osteosynthesis of proximal tibial fractures may be allowed. Dan Med J.

[23] Raschke MJ, Kittl C, Domnick C (2017). Partial proximal tibia fractures. EFORT Open Rev.

[24] Williamson M, Iliopoulos E, Jain A, Ebied W, Trompeter A (2018). Immediate weight bearing after plate fixation of fractures of the tibial plateau. Injury.

[25] Higgins TF, Kemper D, Klatt J (2009). Incidence and morphology of the posteromedial fragment in bicondylar tibial plateau fractures. J Orthop Trauma.

